# Editorial: Insulin resistance and cardiovascular disease

**DOI:** 10.3389/fendo.2023.1266173

**Published:** 2023-08-04

**Authors:** Alan J. Stewart, Erkan Tuncay, Samantha J. Pitt, Richard D. Rainbow

**Affiliations:** ^1^ School of Medicine, University of St Andrews, St Andrews, United Kingdom; ^2^ Department of Biophysics, Faculty of Medicine, Ankara University, Ankara, Türkiye; ^3^ Department of Cardiovascular and Metabolic Medicine & Liverpool Centre for Cardiovascular Sciences, Institute of Life Course and Medical Sciences, University of Liverpool, Liverpool, United Kingdom

**Keywords:** diabetes, heart, vascular system, hypertension, hyperglycaemia, insulin resistance

Diabetes affects 425 million individuals worldwide, with numbers projected to increase to >600 million in the next 20 years (1). In type 1 diabetes (T1D), patients experience insulin deficiency caused by reduced insulin production, whereas in type 2 diabetes (T2D), patients experience insulin resistance (IR), which is often associated with obesity (2). The major factors contributing to the development of IR are increased oxidative stress, hyperglycaemia, and elevated lipid levels (3). Despite advances in therapies that help to control blood glucose levels, cardiovascular complications remain a major cause of morbidity and mortality in this population (2, 4, 5). In the heart, IR causes dysregulated calcium handling, mitochondrial dysfunction, and metabolic inflexibility resulting in a range of pathologies that include dysregulated myocardial-endothelial interactions, impaired diastolic dysfunction, myocardial cell death, and fibrosis (6, 7). Vascular events associated with IR are generally related to hypertension and an enhanced thrombotic environment (8, 9). While obstructive blood clots can lead to myocardial infarction, cerebrovascular events, or critical limb ischaemia and occur as the result of complex interactions between platelets and haemostatic proteins (10). The risk of developing such complications is variable across this highly heterogeneous population and is determined by a range of factors including age, duration of diabetes, glycaemic control, and IR. In this Research Topic of Frontiers in Endocrinology we present 8 articles focused on exploring the relationship between IR and cardiovascular health.

Arteriosclerosis is well-known complication of diabetes (11). He et al. examined whether radiomic intermuscular adipose tissue (IMAT) analysis could be used as a diagnostic measure to indicate arteriosclerosis in T2D patients. A total of 549 patients with newly diagnosed T2D were included and carotid plaque burden was used to indicate arteriosclerosis. Three models were constructed to evaluate the risk of arteriosclerosis: a clinical model, a radiomics model (based on IMAT analysis of chest CT images), and a clinical-radiomics combined model (a model that integrated clinical-radiological features). The performance of the three models were compared using the area under the curve and DeLong tests. The clinical-radiomics combined model and radiomics model indicated a better performance than the clinical model in indicating arteriosclerosis. The authors therefore concluded that the radiomics IMAT analysis was useful for indicating arteriosclerosis in patients with newly diagnosed T2D.

Most people with diabetes have hypertension and 17% of individuals with hypertension (blood pressure 140/90 mmHg or greater, or on anti-hypertensive treatment) have diabetes (12). Youseff et al. investigated inflammation, oxidative stress and mitochondrial biomarkers in 384 participants (210 healthy controls, 55 prediabetic patients, 32 T2D, and 87 patients with T2D and hypertension (T2D+HT)). For the transition from prediabetes to T2D, IL-10, C-reactive protein, 8-hydroxy-2’-deoxyguanosine (8-OHdG), humanin, and p66Shc were the most discriminatory biomarkers, generally displaying elevated levels of inflammation and OS in T2D, in addition to disrupted mitochondrial function as revealed by p66Shc and HN. Disease progression from T2DM to T2DM+HT indicated lower levels of inflammation and OS as revealed through IL-10, IL-6, IL-1β, 8-OHdG and oxidized glutathione (GSSG) levels, most likely due to antihypertensive medication use in the T2DM +HT patient group. The results also indicated better mitochondrial function in this group as shown through higher HN and lower p66Shc levels, which can also be attributed to medication use.

On a similar topic, Ren et al. examined the relationship between the visceral adiposity (as measured by the Chinese visceral adiposity index (CVAI)) and comorbidity of hypertension and diabetes in a large cross-sectional study involving 3,316 Chinese participants aged ≥60 years. A linear association between CVAI and hypertension-diabetes comorbidity was identified, and a 45% increased risk was associated per SD increase. Linear associations of CVAI with hypertension or diabetes, hypertension alone, and diabetes alone were also reported, and the risk increased 39%, 36%, and 28%, respectively per SD increment. Mediation analyses revealed the triglyceride-glucose (TyG) index, an established marker for IR, played a role in the associations suggesting that IR played a mechanistic role in the observed phenomena.

Pan et al. explored the relationship between the TyG index and the incidence of hypertensive disorder of pregnancy (HDP) and adverse pregnancy outcomes. As part of this study 289 women with HDP and 861 women without HDP were recruited in a case-control study. The TyG index was positively associated with HDP incidence, systolic blood pressure (SBP), and diastolic blood pressure (DBP) levels one week before delivery as well as low birth weight and foetal distress incidence. Spline regression revealed a linear correlation between HDP incidence and early trimester TyG index when this value was >8.5. This work highlights a novel link between IR and HDP and adverse outcomes of pregnancy.

In addition to hypertensive disorders, the relationship between TyG and other cardiovascular outcomes was explored in this Research Topic. Sun et al. examined the prognostic potential of the TyG index in patients with ischemic heart failure (HF) after percutaneous coronary intervention (PCI). In the study 2,055 patients with ischaemic HF were retrospectively enrolled and classified into quartile groups based on the TyG index. The primary endpoint was major adverse cardiovascular events (MACE) consisting of all-cause mortality, non-fatal myocardial infarction, and any revascularisation. The incidence of MACE was significantly higher in participants with a higher TyG index, this positive association was non-linear. Furthermore, no obvious interaction was found in the association of TyG with MACE between diabetes group and non-diabetes groups. This work highlights the role of IR in MACE in patients with ischemic HF after PCI.

In a related study, the relationship between TyG index and the prognosis of patients with acute coronary syndrome (ACS) without diabetes who underwent emergency PCI with drug-eluting stents (DESs) was explored by (Zhang Yo. et al.). In this study 1,650 patients were recruited and split into two groups based on the TyG index. The frequency of major adverse cardiovascular and cerebrovascular events (MACCEs; which included all-cause death, non-fatal myocardial infarction (MI), non-fatal ischemia stroke, ischaemia-driven revascularisation and cardiac rehospitalisation) was calculated and compared between the two groups. The high TyG index (≥7.08) group had a considerably greater incidence of MACCE, cardiac death and ischemia-driven revascularization than the low TyG index (<7.08) group. No differences were observed in all-cause death, non-fatal MI, non-fatal ischaemic stroke or cardiac rehospitalisation.

Although multifactorial interventions to control blood glucose, blood pressure, and lipid profiles reduce macrovascular complications and mortality in patients with T2DM, the link between these risk factors has not been fully explored. Zhang Yi et al. recruited 1,920 people across four cities in Anhui Province, China with a mean age of 57.10 ± 10.0 years old. Latent category analysis was used to explore the clustering mode of health risk behaviours (HRBs). The primary exposure was HRBs and exercise and diet interventions, and the primary outcome was cardiovascular disease (CVD) and other variables, including standardized multivariate score (zMS), triglyceride-glucose index (TyG), TyG-WC (waist circumference), TyG-BMI, TG/HDL, and cardiovascular health (CVH). Overall, CVD was found to affect approximately 19.9% of all persons with T2DM. Macrovascular complications of T2D includes coronary heart disease, MI, cardiac insufficiency, and cerebrovascular disease. Elderly age, no occupation, medium and high socioeconomic status (SES), higher level of TyG-WC, and higher zMS (χ2 = 7.59) were correlated with high incidence of CVD. There was a dose−response relationship between HRB co-occurrence and clustering of HRBs and zMS. From an intervention management perspective, exercise and no diet intervention were more significantly associated with CVD; moreover, there was an association between intervention management, gender, zMS, TyG-WC, TyG-BMI, TG/HDL, and CVD. Finally, there was an association between sex, CVH, and CVD. The study highlighted the potential benefits of scaling up multifactorial and multifaceted interventions to prevent CVD in patients with T2DM.

Finally, Macvanin et al. provide a narrative review on the topic of microRNAs and long non-coding RNAs in diabetic cardiomyopathy. The review, which incorporates both animal and human studies, provides compelling evidence to suggest that non-coding RNAs regulate diabetic cardiomyopathy-related processes such as IR, cardiomyocyte apoptosis and inflammation. Emphasis is also placed upon their cardio-protective effects and their potential for diabetic cardiomyopathy treatment is explored.

Collectively, these articles successfully explore the mechanisms by which IR contributes to atherosclerosis, hypertensive disorders, heart disease and metabolic syndrome. In summary, this Research Topic highlights novel associations between IR and health outcomes that may lead to new treatments. In addition, new indices and markers are presented that may serve as potential tools to enable better management of diabetes and its associated complications.

## Author contributions

AS: Writing – original draft. ET: Writing – review & editing. SP: Writing – review & editing. RR: Writing – review & editing.
